# Intravenous iron and iron deficiency anemia in patients with gastrointestinal cancer: A systematic review

**DOI:** 10.1371/journal.pone.0302964

**Published:** 2024-05-22

**Authors:** Shankavi Nandakumar, Navreet Singh, Alliya Remtulla Tharani, Maya Pankiw, Christine Brezden-Masley

**Affiliations:** 1 Department of Medicine, Mount Sinai Hospital, Toronto, ON, Canada; 2 Sinai Health System, Lunenfeld-Tanenbaum Research Institute, Toronto, ON, Canada; 3 Department of Medicine, Temerty Faculty of Medicine, University of Toronto, Toronto, ON, Canada; Medizinische Fakultat der RWTH Aachen, GERMANY

## Abstract

**Background:**

Iron deficiency anemia (IDA) is a prevalent hematological complication associated with gastrointestinal (GI) cancers due to an increased loss of iron and decreased iron absorption. The purpose of this systematic review is to evaluate the use of parenteral iron to treat IDA in patients with GI cancer.

**Methods:**

PubMed, Cochrane, EMBASE, CINHAL and Scopus were searched from January 1, 2010 to September 29, 2023 with no language restrictions. We excluded editorials, case reports, abstracts, conference papers, and poster presentations. Studies were included if they discussed IDA, GI neoplasms, use of iron supplementation (with or without erythropoietin-stimulating agents [ESAs]), defined anemia and had an adult patient population. We assessed the efficacy of parenteral iron in comparison to other iron supplementation methods when treating IDA in patients with GI cancer. The Cochrane Risk of Bias Tool 2 (RoB 2) and the Risk Of Bias In Non-randomized Studies of Interventions (ROBINS-I) assessment tools were used to assess the quality of the included studies. Moreover, the Cochrane Effective Practice and Organization data collection form was used to collect pertinent study information.

**Results:**

Our search yielded 3,969 studies across all databases. Twenty-one studies were included (6 randomized control trials; 15 non-randomized studies). Of the 15 studies evaluating hemoglobin (Hb) response, seven studies found an increase in Hb levels when patients were treated with IV iron. The 14 studies evaluating red blood cell (RBC) transfusion rates found conflicting differences in RBC transfusion needs when treated with IV iron. Studies analyzing health related outcomes typically found an increase in quality of life and decreased post-operative complications.

**Discussion:**

This review demonstrates improved outcomes of IDA in patients with GI cancer treated with IV iron instead of other iron supplementation methods. Timely diagnosis and appropriate IDA management can greatly improve quality of life in this patient population, especially if myelosuppressive chemotherapy is required.

## Introduction

Luminal gastrointestinal (GI) cancers (i.e., cancers of the esophagus, stomach, large and small intestine) represent 26% of global cancer incidence, and 35% of all cancer-related deaths [1]. A common hematological complication associated with luminal GI cancers both at diagnosis and during treatment is anemia, defined by the World Health Organization (WHO) as hemoglobin (Hb) of less than 12 g/dL in women, and less than 13 g/dL in men [2, 3]. The cause of anemia in cancer patients is multifactorial in nature, and can be attributed to comorbidities such as bleeding, hemolysis and nutritional deficiencies [4], such as iron deficiency. The prevalence of iron deficiency anemia (IDA; defined as anemia associated with iron deficiency) in patients with GI cancer ranges from 7% to 42% [5].

The etiology of IDA in patients with GI cancer can be attributed to increased loss of iron (i.e., bleeding) and decreased iron absorption [6]. Systemic inflammation caused by GI malignancy can lead to increased levels of hepcidin, which in turn inhibits iron absorption in the GI tract and iron release from bodily stores [7]. Cytokines associated with systemic inflammation characteristic of GI malignancy also directly inhibit erythropoietic activity, which in combination with poor iron absorption and release, may further worsen IDA [7, 8]. In addition, myelosuppressive chemotherapy and/or radiotherapy can impair normal hematopoiesis, decreasing systemic iron utilization, directly resulting in anemia [7, 8]. Ultimately, multiple factors contribute to IDA associated with GI cancer, contributing the overall difficulty of treating the condition in this patient population.

In patients with GI cancer, IDA is associated with increased risk of mortality, poor response to anticancer treatment, lower overall and progression free survival, fatigue, and poor quality of life (QoL) [9–12]. Targeted treatment of anemia in patients can both improve prognosis and QoL [13]. Common treatment approaches in this patient population include red blood cell (RBC) transfusions, erythropoietin stimulating agents (ESA) and iron supplementation. Despite available treatment options, approximately 60% of patients with anemia do not receive any treatment [2]. Potential reasons for patient undertreatment may include ‐ poor optimization of care across the patient journey, unclear guidelines, and inadequate testing for anemia and IDA [2].

Treatment of anemia with RBC transfusions should be used cautiously as use is associated with increased risk of morbidity and mortality in cancer patients [14]. ESAs offer a means of reducing the need for RBC transfusions but only 30–75% of patients may respond to treatment and use may increase the risk of thromboembolic events [14]. Furthermore, intravenous/parenteral (IV) iron alone or in combination with ESAs presents itself as an effective treatment for anemia, while also reducing the need for RBC transfusions [15]. Oral iron supplementation is also commonly prescribed to address IDA in the cancer population; however, evidence suggests oral iron does not reduce the risk of RBC transfusion, most likely due to either malabsorption of oral iron, non-adherence, slow bioavailability and repletion [16].

The 2018 European Society for Medical Oncology (ESMO) clinical practice guidelines [17] recommend that RBC transfusions only be used in patients with severe anemia-related symptoms, and ESAs only be employed when patients undergoing chemotherapy have had their iron deficiency corrected. Additionally, the American Society of Clinical Oncology (ASCO)/American Society of Hematology (ASH) recommends that ESAs be offered only to patients whose cancer treatment is not curative in intent and with Hb <10 g/dl [18]. However, this recommendation is based on a lack of evidence indicating whether a particular patient population receiving ESAs is at greater or lesser risk of harm particularly in terms of progression/reoccurrence and overall survival [18]. The current lack of specific guidelines leaves physicians without clear directives for treating IDA in patients with GI cancers. Moreover, the existing general guidelines [17, 18] do not advise on the use of parenteral iron supplementation, with or without ESAs, nor do they address the appropriate timing for administering treatment to patients. In addition, existing systematic reviews predominantly focus on the use of parenteral iron supplementation for treating chemotherapy-induced anemia, and the addition of parenteral iron to ESAs in cancer patients more broadly [16, 19, 20]. To our best knowledge, no systematic review exists to date, that examines the use of IV iron in patients with GI cancers with respect to when patients are being diagnosed with IDA, when they are being treated, how they are optimally treated, and the benefits of treatment.

Therefore, given the heightened prevalence of IDA in this patient population, the purpose of this systematic review is to evaluate the use of IV iron to treat IDA in patients with GI cancer.

## Methods

This systematic review was performed following the Cochrane Training Handbook guidelines, as well as the Preferred Reporting for Systematic Reviews and Meta-Analyses (PRISMA) 2020 checklist [21, 22]. This review was not registered, and the review protocol is not available.

### Search strategy

A search using the following databases: PubMed, Cochrane, EMBASE, CINHAL, and Scopus was conducted. The search terms included but were not limited to: “iron deficiency”, “anemia”, “gastric cancer”, “ESA therapy*”, “intravenous iron” and “iron studies”. Randomized control trials (RCTs), systematic reviews, observational studies, case studies, and cohort studies, from January 2010 to September 2023, with no specified language restrictions were retrieved. Studies were excluded if they were editorials, case reports, abstracts, conference papers or poster presentations.

### Study selection

Studies were reviewed if they included the following: 1) iron deficient anemia, 2) gastrointestinal neoplasms, 3) iron supplementation alone (i.e., intravenous iron or parenteral iron, oral iron) or in conjunction with ESAs, 4) defined anemia and the symptoms associated with anemia, and 5) an adult (≥ 18 years of age) population. Additionally, literature published before 2010 was excluded from this systematic review as the use of large dose, IV iron formulations like ferric carboxymaltose (FCM) and iron isomaltoside became readily available for the correction of anemia in 2010 [23].

The web-based software, Covidence^TM^, was used by two authors (SN and NS) to screen studies and extract data [24]. A standardized eligibility checklist was used to screen the title, abstract and full-text of studies; removing ineligible studies throughout the process. Any conflicts that arose were discussed and resolved between the authors (SN and NS).

### Data collection and quality assessment

The Cochrane Effective Practice and Organization data collection form was used to curate a standardized extraction sheet to collect the study information needed from each extracted article [21].

The Cochrane Risk of Bias Tool 2 (RoB 2) was used to assess risk of bias among eligible RCTs [25]. RCTs were assessed and given an overall judgement of low risk-, some concerns-, or high risk of bias [25]. Moreover, the Risk Of Bias In Non-randomized Studies of Interventions (ROBINS-I) tool was used to assess the quality of non-RCT studies [26]. The non-RCT studies were given an overall judgement of low, moderate, serious, or critical risk of bias [26]. The risk assessment for this study was completed individually by reviewers (SN and NS). Visualization of risk-of-bias assessments were generated using the *robvis* online tool [27].

### Statistical analysis

Statistics used in the study were expressed as means, medians, standard deviations (SDs), interquartile ranges (IQRs), and 95% confidence intervals (CI) for any relevant study variables.

## Results

**Fig 1:**
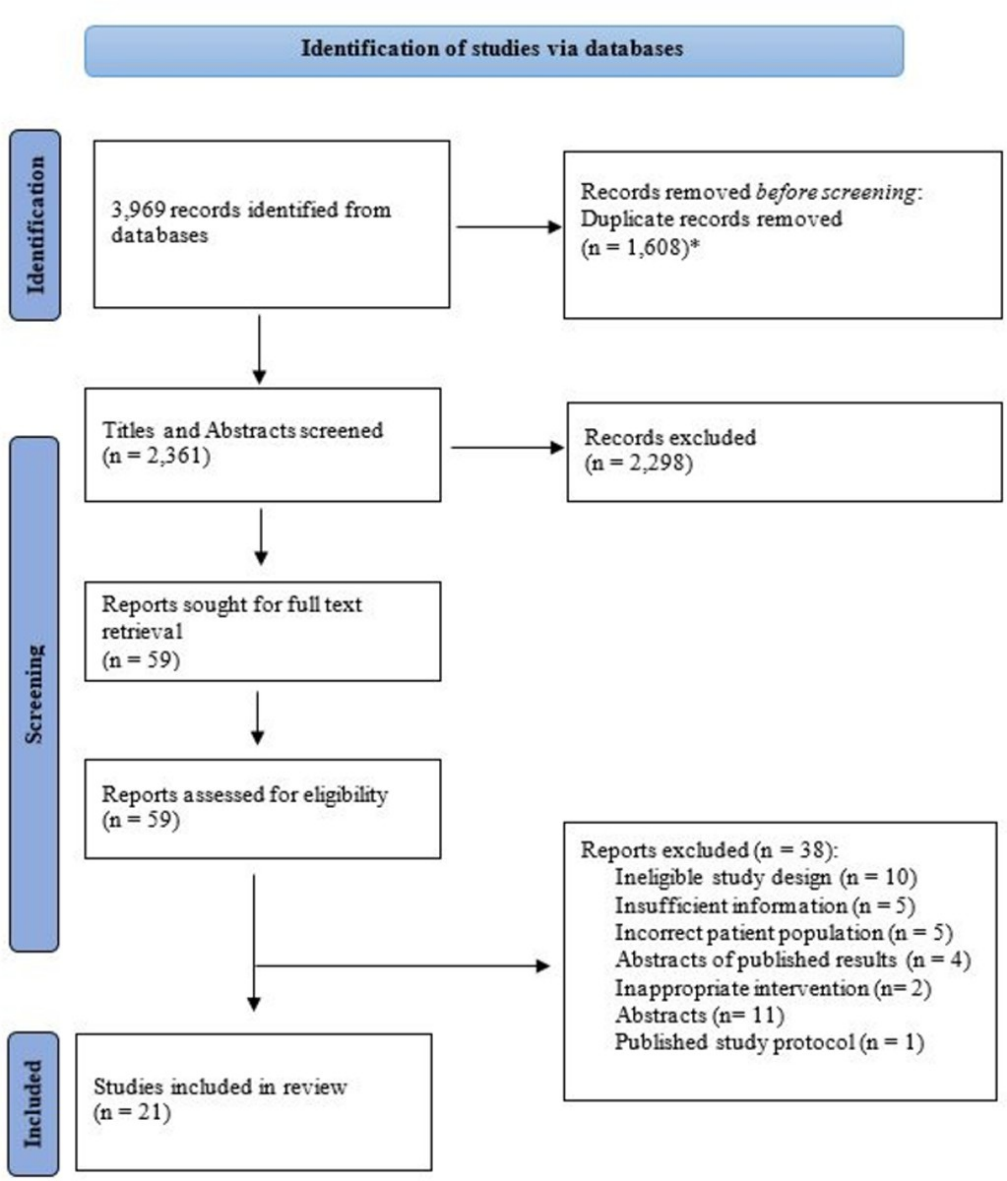
PRISMA diagram for study identification, screening, and inclusion. * Duplicates removed by software program.

Twenty-one studies published between January 1, 2010 and October 1, 2023 met the inclusion criteria and were included in the final analysis. A summary of the screening process can be found in Fig 1. Of these studies, six RCTs compared IV iron supplementation to standard of care or compared two IV iron interventions to one another. The remaining 15 studies were of non-randomized design (10 retrospective, 5 prospective).

All studies included a comparator arm, except for Bojesen et al. [28], Lima et al. [29] and, Verhaeghe et al. [30] where all patients received IV iron. Of the RCTs, Keeler et al. [31], Keeler et al. [32], and Talboom et al. [33] compared ferric carboxymaltose (FCM) to oral iron, while Laso-Morales et al. [34] compared FCM to iron sucrose (IS), and Ng et al. [35] compared iron isomaltoside to standard of care. The comparator arms in 11 studies [36–46] were patients who received no specific treatment or oral iron. Of note, Quinn et al. [47] compared the efficacy of a new anemia management intervention in which patients were given FCM, and then retrospectively compared to pre-intervention patients. IV iron treatment was provided preoperatively in 17 studies and postoperatively in four studies. A complete summary of the studies can be found in Tables 1 and 2.

**Table 1:** Demographic characteristics of included studies.

**Randomized Control Trials**							
**Study (Author, Year)**		**Design**		**Type of Cancer**		**Treatment Arms (if applicable)**		**Sample size (N)**	**Iron type and dose**		**Age, years (Mean ± SD)**		**Setting**	
**(Group 1; Group 2)**
Fung, 2022			RCT			Colorectal Cancer			IV iron isomaltoside		20		Patients received a dose of IV isomaltoside (20 mg.kg^-1^ up to 1000 mg infused over 30 minutes) three weeks before scheduled colorectal surgery	68.4 ± 6.8		Preoperative inpatient setting.		
Patients received an additional dose if Hb < 10 g/dL
Standard care (control)	20	Received usual preoperative care (no treatment) three weeks before scheduled colorectal surgery	69.8 ± 12.6
Keeler, 2017				RCT				Colorectal Cancer				IV FCM		55		Hb>10 g/dL were given 1000 mg (1 dose) of IV FCM if they were <70 kg. If more than 70 kg, they were given 1500 mg (2 doses) of IV iron	Median (IQR):	Preoperative patient setting
Patients that had Hb <10 g/dL and <70 kg got 1500 mg (2 doses) and >70 kg got 2000 mg (2 doses)	73.8 (67.4–78.6)	Patients were given an initial dose of IV iron a minimum of 14 days before planned surgery. Patients receiving oral iron received FS, twice daily until surgery		
FS (Oral iron)		61		Patients received 200 mg of FS, twice daily from initial recruitment until surgery		Median (IQR):
74.7 (67.9–80.8)
Keeler, 2019				RCT				Colorectal Cancer				IV FCM			55			Patients received IV FCM.	73.8 ± 8.9			Preoperative inpatient setting	
Dosage was given based on weight and Hb levels	
IV FCM was dosed by weight and Hb levels. FS was given twice daily until surgery.	
FS (Oral iron)	61	Patients received 200 mg of FS, twice daily until surgery	76.5 ± 10.9
Laso–Morales, 2021			RCT			Colorectal Cancer			IV FCM		50		A single 1000 mg dose of IV FCM in 15 minutes		73 ± 10		Postoperative outpatient setting
Patients were given a single dose of IV FCM a day after surgery. IV IS was given to repeated doses every 48 hours from the day after surgery until hospital discharge or until total dose equivalent to iron deficit, whichever first	
IV IS	51	200 mg of IV IS every 48 hours from POD1 up to hospital discharge or up to the total dose equivalent to the iron deficit (cumulative dosage is calculated depending on patient’s weight and Hb levels)	71 ± 12
Ng, 2018				RCT				Esophagogastric adenocarcinoma				IV iron isomaltoside		11		Patients received a single dose of iron isomaltoside 1000 mg diluted in 250 mL 0.9% sodium chloride and infused over a period of 60 minutes		Median (range):	Perioperative oncology setting			
69 (48–85)
Standard care (control)		13		Had anemia treated with traditional regimens as decided by the clinical oncology team		Median Range:
68 (38–79)
Talboom, 2023				RCT				Colorectal cancer				IV FCM		96		Patients received a maximum of 1000 mg of IV FCM over 15 minutes. If the patient required more than 1000 mg of IV FCM, they would receive the remainder dose at least a week apart from the first dose		Median (range):	Preoperative inpatient setting
72 (63–79)	Patients with severe anemia (Hb ≤ 6.2 mmol/L) received 1500 mg if weight was 35–70 kg and 2000 mg if more than 70 kg. Patients with mild anemia (Hb > 6.2 mmol/L) received a dose of 1000 mg if weight was 35–70 kg and 1500 mg if more than 70 kg. (Hb > 6.2 mmol/L).	
FS (Oral iron)		106		Patients received three 200 mg ferrous fumarate tablets daily from randomization until the day before surgery		Median Range:
70 (61–81)	Patients received oral ferrous fumarate three times daily from randomization until the day before surgery
**Observational Studies**							
**Study (Author, Year)**		**Design**		**Type of Cancer**		**Treatment Arms (if applicable)**		**Sample size (N)**	**Iron type and dose**		**Age, years (Mean ± SD)**		**Setting**	
**(Group 1; Group 2)**
Bojesen, 2021			Observational Cohort Study			Colorectal Cancer			Responders to IV iron isomaltoside (>0.64 g/dL increase in Hb during treatment)	50	Patients weighing <50 kg were given a single total dosage of calculated by the following Ganzoni formula: total dosage (mg Fe) = patient weight (kg) × [target Hb value (g/dl)–current Hb value (g/dl)] × 2.4 + reserve Fe (500 mg) (With a maximum of 20 mg/kg)		71 ± 9.6	Preoperative inpatient setting
Poor responders to IV iron isomaltoside (<0.64 g/dL increase in Hb during treatment)		12		76 ± 12.1		Surgery was planned approximately 4 weeks after iron administration	
If cumulative dosage exceeded 20 mg/kg, a second infusion with the remaining dosage was given a week after the initial dosage
Kam, 2020					Observational Single Center Study					Colorectal Cancer					IV IS OR IV iron isomaltoside			38			Patients were either given 500 mg of IS mixed with 250 ml normal saline over 210 minutes with a total of two doses set at 1 week apart	Median (range):	Preoperative inpatient setting
OR	70.5 (45–85)		IV iron doses were given at least 2 weeks prior to their elective operation date			
1000 mg of iron isomaltoside in 100 mL of normal saline over 15 minutes as a single dose
No iron therapy		62		The non-IV iron treatment group was the control group for this study		Median (range):
69 (43–88)
Laso-Morales, 2017					Retrospective Observational Study					Colorectal Cancer					IV IS or FCM				232				Cumulative IV iron dosage (mg) was calculated using—14 –baseline Hb x 2.4 x body weight kg + 500	71 ± 11				Preoperative inpatient setting		
Patients received either IV IS or IV FCM.
Patients who received IV IS were given doses of 200 mg in 100 mL saline over 30–60 minutes. These doses were given up to three times a week preoperatively. Patients receiving IV FCM were given as 500–1000 mg doses in 200 mL saline over 15 to 30 minutes, once a week preoperatively	
Patients were given IV IS three times a week preoperatively until total iron dosage was given or they were given IV FCM one a week preoperatively until total iron dosage was given	
Oral iron or no iron treatment	90	Patients were either given no iron (if they were not referred to the anemia clinic) or given 100 mg of elemental iron per day (if they had contraindication to receive IVI)	69 ± 15
Laso-Morales, 2018					Retrospective Observational Single-Center Study					Colorectal Cancer					IV IS				47				Cumulative IV iron dosage was calculated depending on the patients baseline Hb and bodyweight	72 ± 10.2				Postoperative outpatient setting	
For patients that did not receive IV iron pre-operatively, 500 mg IV iron were added for deposits	
Patients receiving IV iron were given doses three times a week during hospitalization until total iron dosage was given. Patients receiving oral iron were given 100 mg iron per day on discharge		
Iron sucrose was given at doses of 200 mg in 100 mL saline over 30–60 minutes, up to 3 times a week during hospitalization
Oral iron (elemental iron)	92	Patients that did not receive the total IV iron calculated dose during hospitalization were given 100 mg of elemental oral iron on discharge	69 ± 11
Lima, 2018			Observational Single Center Study			Colorectal Cancer			All patients received at least one dose of IV FCM			30			During the study period (12–14 weeks), in every visit, the need of continuation of iron replacement therapy was evaluated and, if necessary, patients were treated with IV FCM, according to the approved summary of product characteristics, until anemia or iron deficiency was corrected		Median (IQR): 67.0 (60.0–75.8)			Perioperative oncology setting
During the course of the study period, the need of continuous iron replacement therapy was evaluated, and patients were treated with FCM if needed	
Throughout the study, 25 patients were administered 1000 mg of IV FCM and 5 patients received two administrations of 1000 mg IV FCM each
Quinn, 2017		Retrospective Observational Study		Colorectal Cancer		Post intervention cohort (IV FCM)	76	IV FCM administration done at least 10 days before surgery. 1000 mg over a 15-minute period	61.9 ± 13.9	Preoperative colorectal unit setting	
Pre-intervention cohort	76	The pre-intervention cohort was the control group for this study	68.1 ± 12
Wilson, 2018				Retrospective cohort study				Colorectal Cancer				IV FCM or IV iron maltoside		102		Patients received a single dose of 1000–2000 mg of FCM or iron III maltoside		Median (IQR):	Preoperative surgical clinic setting
75.0 (67.0–80.0)	Received IV iron therapy less than 6 weeks before surgery		
No IV iron		218		The non-IV iron treatment group was the control group for this study		Median (IQR):
73.5 (66.0–80.0)
Wilson, 2018		Retrospective cohort study		Colorectal Cancer		IV FCM or IV iron maltoside	94	Patients received a single dose of 1000–2000 mg of FCM or iron III maltoside	71.8 ± 11.1	Preoperative surgical clinic setting	
No IV iron	224	The non-IV iron treatment group was the control group for this study	73.7 ± 9.9
Calleja, 2016			Multicenter cohort study			Colon Cancer			IV FCM		111		IV iron group received a median total FCM dose of 1000 mg, and the administration was done on average 28.5+/- 16.7 days before surgery		72.9 ± 11.1		Preoperative inpatient setting
IV FCM was given to patients 2–4 weeks before scheduled surgery	
No IV iron treatment	155	No-IV group received different doses and formulations of oral iron supplementation at the time of diagnosis	70.8 ± 10.3
Kangaspunta, 2022				Retrospective cohort study				Colon Cancer				IV FCM			180			Patients in this group received either 500 mg or 1000 mg of IV FCM at a time (also dependent on patient’s weight and Hb levels)	Median (IQR): 73.8 (66.9–80.1)			Preoperative outpatient setting	
Forty patients received 500 mg, 1 patient received 800 mg, 109 patients received 1000 mg, 1 patient received 500 mg + 500 mg, 9 patients received 1000 mg + 500 mg, 9 patients received 1000 mg + 1000 mg of IV iron, and 1 patient received 500 mg + 1000 mg + 1000 mg.	
Patients were given preoperative IV iron up to 60 days prior to operation	
No IV iron treatment	138	The non–IV iron treatment group was the control group for this study	Median (IQR): 76.0 (70.1–81.9)
Titos-Acros, 2021			Retrospective Observational Study			Colon Cancer			Postoperative IV iron saccharose		52		Administered dose was 100–200 mg IV iron III saccharose (maximum dose: 200 mg three times a week)		70.9 ± 11.1		Postoperative surgical clinic setting
Patients receiving IV iron received a maximum of three doses a week until discharge	
No postoperative IV iron saccharose	52	The non-IV iron treatment group was the control group for this study	70.6 ± 10.9
Jeong, 2014					Observational Study					Gastric carcinoma					IV IS				68				300 mg IS made up to 250 ml with 0.9% saline and repeated until the total target amount was given	63.2 ± 11.2				Postoperative outpatient setting	
The total iron (mg) to be administered was calculated as:	
Patients received IV iron every other day until total target amount was given to each patient		
body weight (kg) 9 (target Hb (12)- patient’s Hb) 9 x 0.24 + 500 mg
No specific treatment	74	The non-IV iron treatment group was the control group for this study	62.6 ± 11.7
Verhaeghe, 2017	Retrospective Observational Study	Gastrointestinal malignancies	All patients received at least one dose of IV FCM	303	FCM administration was done at baseline. Most patients only received one dose of FCM (if patients were given another dose within four weeks, the two doses were considered one cumulative dose)	63.22 ± 11.91	Preoperative digestive oncology clinic setting
Ploug, 2022		Retrospective Cohort Study		Colorectal cancer		IV iron isomaltoside	122	Patients received a single dose of IV iron isomaltoside. The modified Ganzoni formula was used to determine the total iron dosage per person. The maximal dose to be administered was 20 mg/kg	Median Range: 74.5 (63.5–82.5)	Preoperative inpatient setting	
No IV iron treatment	48	The non-IV iron treatment group was the control group for this study	Median Range: 75.0 (70.0–82.0)
Ploug, 2023			Retrospective Cohort Study			Colorectal cancer			IV iron isomaltoside		89		Patients received a single dose of IV iron isomaltoside. The modified Ganzoni formula was used to determine the total iron dosage per person. The maximal dose to be administered was 20 mg/kg		Median Range: 73.0 (63.0–80.0)		Preoperative inpatient setting
Patients received a single dose of IV iron nine days before colorectal surgery	
No IV iron treatment	36	The non-IV iron treatment group was the control group for this study	Median Range: 75.0 (80.0–82.0)

**Table 2:** Main results of included studies.

Randomized Control Trials					
Study (Author, Year)	Treatment Arms (if applicable)	Diagnostic Criteria	Outcomes of Interest	Results	Conclusion
Fung, 2022	IV iron isomaltoside vs. Standard care (control)	Hb <13 g/ dL for all participants Ferritin <30 ng/L OR 30 to 100 ng/L with TSAT <20%	Hb levels	The mean Hb change from baseline to before surgery was higher in the IV iron group than the control group (mean difference of 6.1 g/dL, p = 0.040)	IV iron had significant impacts on the perioperative changes of Hb, ferritin, and TSAT concentrations in IDA patients with colorectal cancer
			Blood transfusion rates	The number of patients needing RBC transfusions during the perioperative period was 2 in the IV iron group and 6 in the control group (p = 0.235)	
			Iron parameters (Serum ferritin, TSAT)	Ferritin level changes from baseline to before surgery was largely in favour of the IV iron group (mean difference of 296.9 μg/L, P<0.001)	
				The TSAT concentration was higher in the IV iron group compared to the control group with a mean difference of 4.8% (P<0.001)	
Keeler, 2017	IV FCM vs.FS (Oral Iron)	Hb <11g/ dL in women Hb <12g/dL in men	Hb levels	Increases in Hb were higher in IV iron recipients (median 1.55 g/dL [IQR 0.93–2.58] vs. 0.50 g/dL [IQR -0.13 to 1.33]; p<0.001). Hb levels were higher at surgery after treatment with IV iron than oral iron	IV iron did not reduce blood transfusion needs but was more effective than oral iron at treating preoperative anemia and iron deficiency
			Blood Transfusion Rates	No difference in blood transfusion needs from recruitment to trial completion in terms of volume of blood administered (p = 0.841) or number of patients transfused (p = 0.470).	
			Iron parameters (Serum ferritin, TSAT)	Ferritin levels were significantly higher in the intravenous group at surgery (median 558 (IQR 330–1085) μg/l versus 27⋅5 (17–51⋅5) μg/l in oral group; P <0⋅001)	
				This same relationship was evident with TSAT levels at surgery (median 19 (16–29) and 9(5–14) % respectively; P <0⋅001)	
			Postoperative complications	There was no differencein grade of complication severity between groups from recruitment to outpatients (P = 0⋅995) or in complication rate over the same period (P = 0⋅305). The same was true for grade (P = 0⋅083) and rate (P = 0⋅091) of infective complications.	
			90-day mortality	Ninety-day mortality rates were similar (2 and 3 deaths respectively; P = 0⋅906).	
			Postoperative length of hospital stay	Postoperative length of hospital stay was 6 days for both groups (IQR 4–9 and 5–10 days for oral and intravenous groups respectively; P = 0⋅950)	
Keeler, 2019	IV FCM vs. FS (Oral Iron)	Hb <11g/ dL in women Hb <12g/dL in men	Hb levels	Hb levels were higher at surgery and in outpatients with IV iron however, no difference in blood transfusion use was seen	IV iron was more efficacious at improving QoL scores than oral iron
				Eleven QoL components increased by a clinically significant margin in the IV iron group between recruitment and surgery with one component for oral iron. Fact-An scores were higher for the IV iron group.	
			QoL		
Laso-Morales, 2021	IV FCM vs. IV IS	Hb <11 g/dL for all patients	Hb levels	No between-group differences in mean change in Hb from postop day 1 to postop day 30 (FC: 2.5 g/dL, 95% CI: 2.1–2.9; IS:2.4g/dL, 95% CI:2.0–2.8; p = -0.52)	Hb increase was similar for both IV FCM and IV IS, but infection rate was higher in IS
			Blood Transfusion Rates	No between-group differences in blood transfusion rates	
			Iron parameters (Serum ferritin, TSAT)	Both ferritin and TSAT levels were higher on the day of surgery than at the time of CRC diagnosis for FCM group	
			Postoperative complications	Infection rate was lower in the FCM (Ferinject™) group compared to IS (Feriv) group (9.8% vs. 37.2%).	
			Postoperative length of hospital stay	No between-group differences in length of hospital stay	
Ng, 2018	IV iron vs. Standard care (control)	Hb <12g/dL in women Hb <13 g/dL in men	Hb levels	No significant change in Hb between both groups with an increase in ferritin levels in IV iron group after cycle one of chemotherapy (Standard care: 116 ng/ml; IV iron 770 ng/ml, P<0.05).	IV iron improves QoL and ferritin levels
			Blood Transfusion Rates	No difference in blood transfusion rates	
			Iron parameters (Serum ferritin, TSAT)	Ferritin showed a significant increase after chemotherapy cycle one in the group treated with IV iron 105 ng/ml to 1015 ng/ml (P<0.05)	
				Transferrin saturations increased above 20% in the intravenous iron group rising from 11.1% to 26.1% after cycle 1 (P = 0.196). Transferrin saturations never exceeded 20% in the standard care group but did rise from 11.9 to 19% after cycle three of chemotherapy.	
			QoL	IV iron group saw improvements in QoL	
			Anemia-specific QoL	IV iron group saw improvements in anemia-specific QoL.	
Talboom, 2023	IV FCM vs. FS (Oral Iron)	Hb <12g/ dL in women Hb <13g/dL in men TSAT< 20%	Hb levels	Mean absolute change in Hb from baseline until admission was similar between IV iron compared with oral iron (0.85 g/dL vs. 0.58 g/dL, p = 0.13)	Changes in Hb levels before surgery were insignificant between both treatment regimens but significantly improved at all other timepoints with the IV iron treatment
			Blood Transfusion Rates	No significant difference in blood transfusion rates between IV iron group and the oral iron group (p = 0.39)	
			Iron parameters (Serum ferritin, TSAT)	Serum TSAT, ferritin, and hematocrit levels were similar at baseline but significantly higher in the IV iron group at day of admission and postoperative timepoints	
			Postoperative complications	The postoperative cumulative complication rate at 6 months was not statistically significant with 48% in the IV iron group and 57% in the oral iron group (p = 0.22)	
			Postoperative length of hospital stay	There was no difference in the total length of stay between IV iron and oral iron groups (p = 0.55)	
**Observational Studies**					
**Study (Author, Year)**	**Treatment Arms (if applicable)**	**Diagnostic Criteria**	**Outcomes of Interest**	**Results**	**Conclusion**
Bojesen, 2021	Responders to IV iron isomaltoside (>0.64 g/dL increase in Hb during treatment) vs. Poor responders to IV iron isomaltoside (<0.64 g/dL increase in Hb during treatment)	Hb ≤11.28 g/dL for all patients	Hb levels	Hb levels improved in patients over the duration of treatment:	Patients with severe anemia (Hb<9.02 g/dL) had the largest increase in Hb after 4 weeks.
				Week 1: 0.77 g/dL (95% CI 0.52–1.03 g/dL, P<0.0001)	
					Patients with mild anemia (>10.31 g/dL) did not have an increase in Hb during the treatment course.
				Week 2: 1.5 g/dL (95% CI 1.21–1.80 g/dL, P<0.0001)	
				Week 4: 2.13 g/dL (95% CI 1.71–2.55 g/dL, P<0.0001)	
			Iron parameters (Serum ferritin, TSAT)	Poor responders had significantly higher ferritin levels at weeks 1, 2 and 4 (P = 0.02, P = 0.003 and P = 0.013, respectively) compared to responders.	
				Poor responders had a significantly lower increase in TSAT 1 week after initial treatment (−11%, 95% CI −18% to −4%, P = 0.0026) compared to responders.	
				No difference in serum iron was found between poor responders and responders at week 1, 2 or 4	
			Postoperative complications	Twenty-one patients (34%) developed a postoperative complication within the first 30 days after surgery. Not responding sufficiently to treatment was associated with higher baseline Hb (P = 0.049) and lower body mass index (P = 0.036) but did not remain significant after correction for multiple testing.	
			Postoperative length of hospital stay	The median length of stay was 3 days (IQR 3–6 days, range 1–36 days).	
Kam, 2020	IV IS OR IV iron isomaltoside vs. No iron therapy	Hb <10 g/dL Hb <12 g/dL after recent transfusion	Hb levels	No statistically significant difference in mean preoperative Hb level between two groups. IV iron group had a higher median Hb rise of 1.9 g/dL vs. 0.6 g/dL in non-IV iron (P<0.001).	Patients with severe anemia (Hb<9.02 g/dL) had the largest increase in Hb after 4 weeks.
					IV iron can significantly increase Hb levels in IDA patients before colorectal surgery and reduce RBC transfusions.
			Blood Transfusion Rates	8 patients in the IV iron group required blood transfusion compared to 30 in the non-IV iron group (P = 0.006).	
			Blood Transfusion Rates	No significant difference in number of patients with red blood cell transfusion prior to IV iron (42.1% in IV iron and 41.9% in non-IV iron, p = 0.987)	
Laso-Morales, 2017	IV IS OR IV FCM vs.Oral iron or no iron	Hb<13 g/dL for all patients	Postoperative complications	There was no difference in RBCT rates between patients with anemia on IV iron and those on standard care (16% vs. 17%; p50.865).	IV iron was more effective in treating preoperative anemia and reducing infection rate than standard of care
				Infection rates were lower among IV iron treated patients (18% vs. 29%; p<0.05). IV iron did not reduce postoperative blood transfusions but was more effective than standard care in treating preoperative anemia.	
			Postoperative length of hospital stay	No significant differences in length of stay	
Laso-Morales, 2018	IV IS vs. Oral iron (elemental iron)	Hb<12 g/dL for all patients	Hb levels	Anemia was more prevalent among patients in the IV iron group (p = -0.027), despite greater increment in Hb (2.0 ± 1.5 g/dL vs. 1.1 ± 1.2 g/dL; p = 0.001).	Compared to standard of care, post-operative IV iron administration improved recovery of Hb levels at 30 days post-operative, without an increase in post-operative complications.
			Blood Transfusion Rates	7 patients in IV iron group and 4 patients in non-IV iron group required postoperative transfusions.	
			Postoperative complications	No differences in post-operative complications between the groups, and no IV iron-related adverse events.	
Lima, 2018	All patients received at least one dose of IV FCM	1) Hb ≤11 g/dL or a reduction ≥2 g/dL since the start of the current chemotherapy regimen AND, 2) Ferritin <30 ng/mL and transferrin saturation (TSAT) <20% OR, Ferritin 30–800 ng/mL, and TSAT<50%	Hb levels	Statistically significant increase in mean Hb (10.3 vs 11.2 g/dL)	FCM was well tolerated and had a positive impact on treatment of IDA
			Iron parameters (Serum ferritin, TSAT)	Ferritin had a mean change of 646.7±455.7 ng/mL (p<0.001)	
				TSAT had a mean change of 6.7%±6.9% (p<0.001)	
				Serum iron had a mean change of 17.4±26.6 mg/mL (p<0.001)	
Quinn, 2017	Post intervention cohort (IV FCM) vs. Pre-intervention cohort	Hb <11g/dL for all patients	Hb levels	Mean day 3 post-operative Hb levels were significantly lower in patients with uncorrected anemia (9.5 g/dL, p = 0.004).	IDA management pathway resulted in improved perioperative Hb levels with reduction in perioperative transfusion.
			Blood Transfusion Rates	Postoperative transfusion rates were 38% in patients with uncorrected anemia, compared to 0% in corrected anemia and 3.5% in patients who were non-anemic.	
			Postoperative complications	There were seven postoperative transfusions in the entire cohort: two in patients who were non-anemic (3.5% transfusion rate), two in patients with iron deficiency (13% transfusion rate) and three in patients with uncorrected anemia from other causes (a 50% transfusion rate).	
			Postoperative length of hospital stay	The difference in length of stay between patients with corrected anemia and patients who were non-anemic was non-significant (p = 0.472).	
			Iron deficiency rates	Treatment resulted in a preoperative IDA correction rate of 60% (nine of fifteen).	
Wilson, 2018	IV FCM or IV iron maltoside vs. No IV iron	Hb < 12.0 g/dL in women Hb < 12.9 g/dL in men	5-year OS	No significant differences in 1-, 3-, and 5-year OS between the IV iron and no IV iron groups	IV iron does not have a profound effect on long-term OS and DFS.
			DFS	No significant differences in DFS between the IV iron and no IV iron group	
Wilson, 2018	IV FCM or IV iron maltoside vs. No IV iron	Hb < 12.0 g/dL in women Hb < 12.9 g/dL in men	Hb levels	In the IV iron group, preoperative Hb level was significantly increased compared to the non-IV iron group (0.65 mmol/L vs. 0.10 mmol/L, p<0.001).	IV iron leads to optimization of preoperative Hb levels
			Blood Transfusion Rates	No significant decrease in the percentage of patients with a postoperative blood transfusion	
			Postoperative complications	No advantageous effect was found on postoperative complications	
Calleja, 2016	IV FCM vs. Oral iron	1) Hb<12 g/dL in women Hb <13 g/dL in men 2) Serum ferritin <30 ng/mL AND/OR 3) Transferrin saturation (TSAT) <20%	Hb levels	Mean total Hb increases significantly favor the FCM group between diagnosis and hospital admission (1.5 vs. 0.5 g/dL; p<0.0001) and between diagnosis and 30 days post-surgery (3.1 vs. 1.5 g/dL; p<0.0001)	Preoperative treatment in patients with colon cancer who were anemic reduced RBC transfusion requirements and length of hospital stays.
			Blood Transfusion Rates	Less patients required RBC transfusion during study in FCM group: 9.9% in treatment group vs. 37% in control group (OR: 5.9, 95% CI: 2.9–11.1, p<0.001)	
			Iron parameters (Serum ferritin, TSAT)	At 30 days after-surgery, the average FCM treated patient presented no recognizable signs of iron deficiency anemia (being the mean of Hb: 12.6 g/dL [≥12 g/dL]; serum ferritin: 218 ng/mL [≥30 ng/mL]; and saturation transferrin index: 25.1% [≥20%]) compared with the no-IV group that did not reach normalized mean values	
			Postoperative complications	A lower number of re-interventions and post-surgery complications were seen in the FCM group (20.7 vs. 26.5%; p = 0.311)	
			Postoperative length of hospital stay	FCM group had a significantly shorter mean length of hospital stay (Mean±SD; 8.4±6.8 days) compared to no-IV iron group (Mean±SD; 10.9±12.4 days)	
Kangaspunta, 2022	IV FCM vs. No IV iron	Hb<12 g/dL in women Hb <13 g/dL in men	Blood Transfusion Rates	No significant difference in number of transfusions	Overall decrease in postoperative complications, and postoperative anemia.
			Postoperative anemia	Patients treated with IV iron had lower prevalence of anemia at 1 month after surgery (38.7% vs. 65.3%, p<0.01) when compared with patients without IV iron treatment.	
			Postoperative complications	Patients treated with IV iron had less post-operative complications (33.9% vs. 45.9%, p = 0.045)	
			30-day and 90-day mortality	No significant difference in mortality	
			Postoperative length of hospital stay	No significant difference in length of hospital stay	
Titos-Acros, 2012	Postoperative IV iron III saccharose vs. No postoperative IV iron	Hb <11g/dL for all patients	Hb levels	Mean Hb at discharge in the IV iron group was 10.0 ± 1.1 g/dL vs. 10.6 ± 1.2 g/dL in control (p = 0.012).	IV iron did not appear to impact blood transfusion requirements
			Blood Transfusion Rates	No significant difference in blood transfusion needs	
Jeong, 2014	IV IS vs. No specific treatment	Hb <10 g/dL for all patients	Hb levels	Mean increase of Hb at 6 months post-operation was 3.2 g/dL in the IV iron sucrose group, and 2.5 g/dL in the no-iron group (P = 0.029).	IV IS significantly increased serum Hb in patients developing acute postoperative anemia after gastrectomy
Verhaeghe, 2017	All study patients received IV FCM	Serum ferritin < 100 μg/L and TSAT < 20% suggested AID	Hb levels	Median Hb change was an increase of 0.5 [IQR; -0.1–1.6] g/dL	Based on the findings, the median increase in Hb over a 28-day period was 0.5 g/dL. This is not a significant difference but may be due to the inadequate dosing schemes. The response to IV iron depends on the underlying mechanism
			Iron parameters (Serum ferritin, TSAT)	Median serum ferritin change was an increase of 464 [IQR; 230–830) μg/L	
				Median TSAT change was an increase of 11 [IQR; 5–18.5] %	
Ploug, 2022	IV iron isomaltoside vs. No IV iron treatment	Hb<12 g/dL in women Hb <13 g/dL in men Ferritin< 50 μg/L	Hb levels	The change in Hb levels between baseline and preoperative was not statistically different between the IV iron group and the control group (p = 0.94)	Preoperative IV iron treatment was not associated with a significant rise in Hb levels at the time of surgery or reduced need for perioperative blood transfusions in IDA patients
			Blood transfusion rates	The transfusion rate from baseline to postoperative day 30 was 45% in the IV iron group and 40% in the control group (p = 0.65)	
			Postoperative complications	The IV iron group had a significantly higher rate of overall postoperative complications (excluding need for blood transfusion) (p = 0.08)	
			Postoperative length of hospital stay	No significant difference in length of hospital stay (p = 0.74)	
Ploug, 2023	IV iron isomaltoside vs. No IV iron treatment	Hb<12 g/dL in women Hb <13 g/dL in men Ferritin< 50 μg/L	Five-year recurrence	The overall five-year recurrence rate of colorectal cancer was 13.5% in the IV iron group and 16.7% in the control group	There is no association between preoperative iron treatment and five-year recurrence rate in colorectal cancer patients with IDA
			Postoperative mortality rates	Postoperative mortality at the follow-up time (4.74–5.00 years) was 13.5% in the IV iron group and 2.8% in the control group	

### Quality assessment

**Fig 2:**
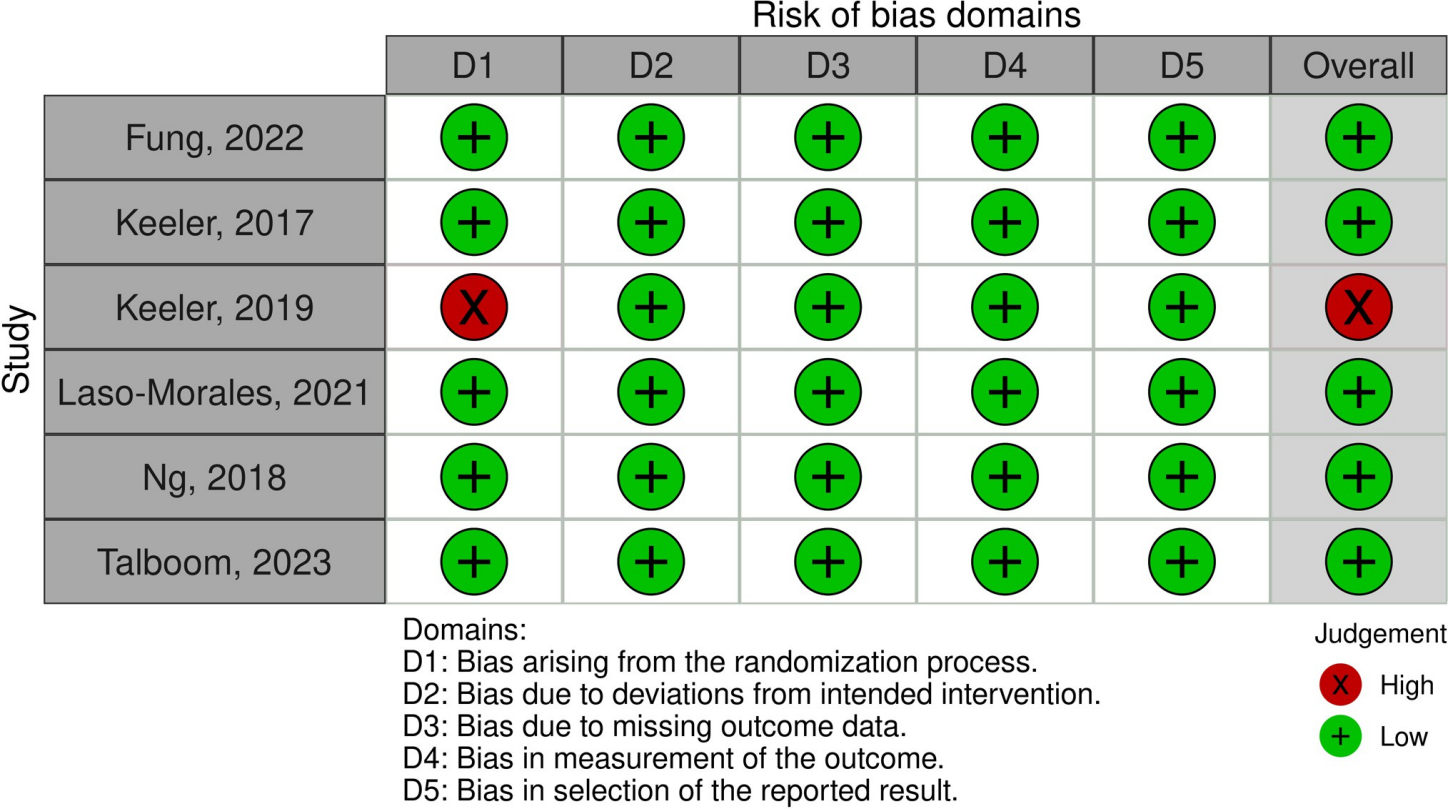
Risk of bias assessment of randomized studies included in the current review.

Five of six randomized trials had a low risk of bias. Keeler et al. [32] had a high risk of bias as patients were not blinded to the study group that they were in. The complete quality assessment of the included RCTs can be found in Fig 2.

**Fig 3:**
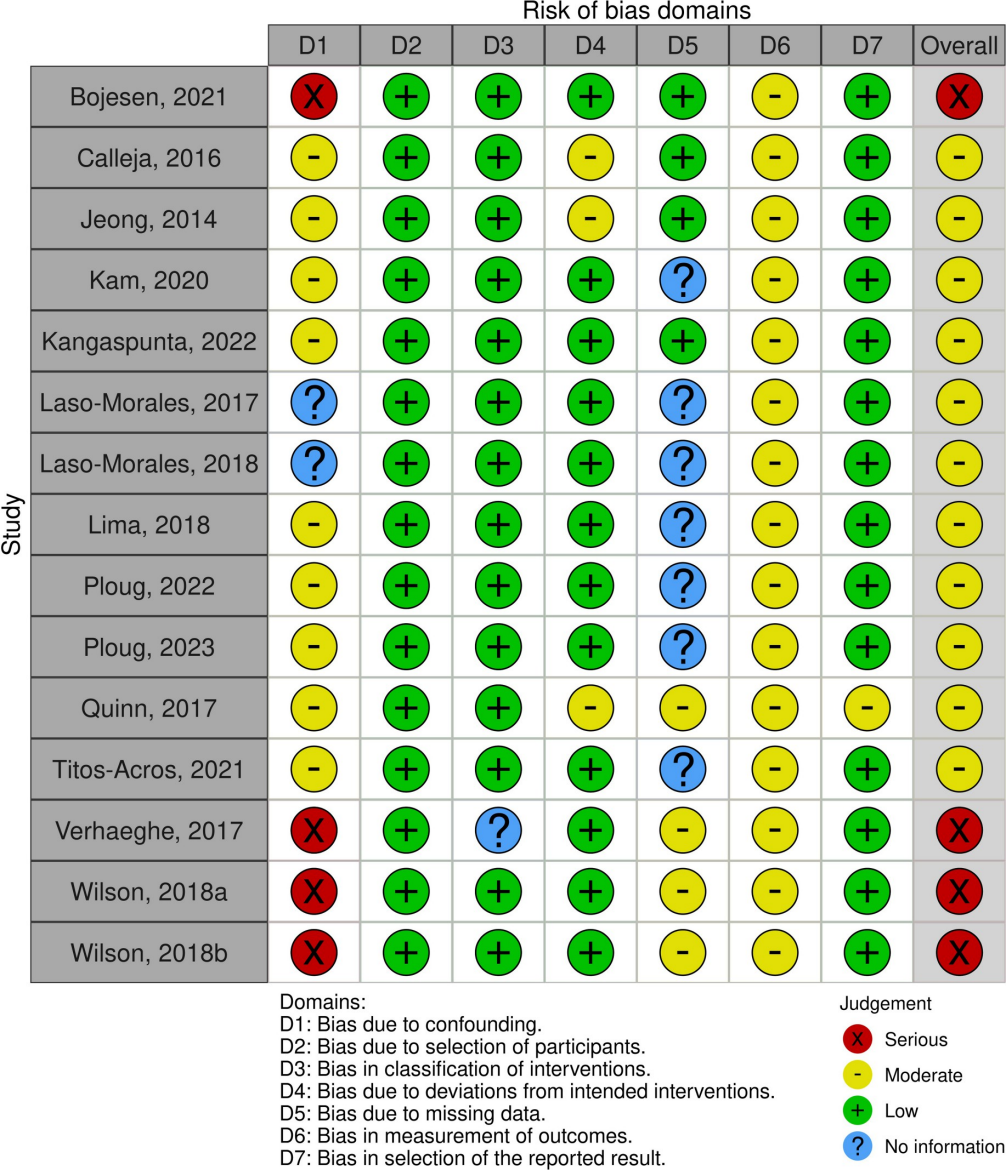
The risk of bias assessment completed for non-randomized studies.

Of the 15 non-randomized studies, 11 studies had a ‘Moderate’ risk of bias, primarily attributable to appropriately controlled baseline confounding factors, missing data, deviations from intended interventions, and lack of information to assess bias within certain domains. Four studies had a ‘Serious’ risk of bias, likely due to the presence of confounding factors without appropriate statistical considerations, as well as a lack of information. A summary of the risk of bias assessment of non-randomized studies can be found in Fig 3.

### Outcomes

Key outcomes were determined *a priori* after examination of relevant literature and considerations of the research question. Three key outcomes were identified: Hb response, RBC transfusion needs, and other applicable health outcomes (e.g., disease free survival (DFS), mortality, morbidity, overall survival (OS), quality of life (QoL), length of hospital stay (LOS), and safety/adverse events).

### Hemoglobin response

All RCTs except Keeler et al. [32], captured Hb response. Fung and colleagues [44] reported a mean difference of 6.1 g/dL (p = 0.040) when comparing patients with colorectal cancer (CRC) who received 20 mg/kg (up to 1000mg) of iron isomaltoside compared to patients who received standard care. Keeler et al. 2017 [31] reported a median increase in Hb of 1.55 g/dL (IQR; 0.93–2.58) in patients with CRC who received 1000 mg or 1500 mg of parenteral FCM at least 14 days prior to surgery compared to an increase of 0.5 g/dL (IQR; -0.13–1.33) in patients who received oral ferrous sulfate twice daily until surgery (*p*<0.001). In the investigation conducted by Laso-Morales et al. [34] patients with CRC either received a single 1000 mg dose of IV FCM on postoperative day two or 200 mg of IV Iron sucrose every 48 hours from postoperative day two to discharge. Patients with esophagogastric adenocarcinoma in the investigation conducted by Ng et al. [35] received a single 1000 mg dose of iron isomaltoside prior to the initiation of chemotherapy or received standard of care. Laso-Morales et al. [34], Ng et al. [35], and Talboom et al. [33] found no significant differences in Hb levels between the intervention and comparator groups.

Of the 15 non-randomized studies, 11 studies assessed serum Hb levels. Bojesen et al. [28] reported a mean increase in Hb of 2.13 g/dL (95% CI: 1.71–2.55 g/dL; *p*<0.0001) after four weeks in patients with CRC who received iron isomaltoside prior to surgery. In addition, Lima et al. [29] reported an absolute increase in mean Hb of 0.9 g/dL (SD 1.3) from baseline to 12–14 weeks (*p* = 0.001) in patients with CRC who received 1000 mg of IV FCM every study visit (12–13 weeks) until anemia or ID was corrected. In contrast, Verhaeghe et al. [30] reported no significant increase in Hb in patients with GI malignancies who received at least one dose of IV FCM preoperatively after a four week follow up period [30].

Two studies comparing IV iron to oral iron reported an increase in Hb. Calleja et al. [36] reported a greater increase in Hb in patients with colon cancer who received IV FCM (median dose of 1000 mg given 28.5 days preoperatively) compared to patients who received varying doses and formulations of oral iron (1.5 g/dL vs. 0.5 g/dL; *p*<0.0001) between diagnosis of anemia and hospital admission for surgery, and between diagnosis and 30 days postoperatively (3.1 g/dL vs. 1.5 g/dL; *p*<0.0001). In addition, the percentage of patients with Hb<10 g/dL was significantly lower in the intervention group at hospital discharge (61.6% vs. 75.7%, *p*<0.05) compared to the oral iron group (36). Laso-Morales et al. [48] reported a greater increase in Hb in patients with CRC who received IV iron sucrose of varying dose compared to patients who received oral elemental iron (2.0±1.5 g/dL vs. 1.1±1.2 g/dL; *p* = 0.001) from postoperative day one to postoperative day 30. However, the prevalence of anemia was greater and more severe in the IV iron group (*p* = 0.027) [48].

Jeong et al. [37], Kam et al. [38], Quinn et al. [47], Titos-Acros [40], Ploug et al. 2022 [46], Ploug et al. 2023 [45], and Wilson et al. [41] compared IV iron to no specific treatment or standard of care. Jeong and colleagues [37] observed a mean increase of Hb of 3.2 g/dL in patients with gastric carcinoma who received IV iron sucrose every other day in 300 mg doses until total target dosage was given (target dosage calculation can be found in Table 1) compared to an increase of 2.5 g/dL in the no specific treatment group (p = 0.029) six months post-operatively (treatment was initiated post-operatively). Kam et al. [38], and Ploug et al. 2022 [46] reported no difference in mean preoperative Hb levels in patients with CRC who received IV iron or no specific treatment. However, the IV iron groups had a higher median Hb rise of 1.9 g/dL vs. a rise of 0.6 g/dL in the no specific treatment group (*p*<0.001). In addition, Quinn et al. [47] reported that mean Hb levels were significantly lower in patients with CRC with uncorrected anemia (no treatment or oral iron) compared to patients with corrected anemia (received 1000 mg of IV FCM) on postoperative day three (9.5 g/dL vs. 10.9 g/dL, *p* = 0.004). Titos-Acros [40] reported mean Hb at discharge was lower in patients with colon cancer who received 100–200 mg of IV iron saccharose postoperatively, compared to those who did not (10±1.1 g/dL, vs. 10.6±1.2 g/dL; *p* = 0.012). Furthermore, Wilson and colleagues [42] reported that patients treated with 1000–2000 mg of IV FCM or IV maltoside less than 6 weeks preoperatively had a significant increase in Hb compared to the usual care group (1.05 g/dL vs. 0.16 g/dL; *p*<0.001).

### RBC transfusion needs

Five randomized studies reported changes in RBC transfusion needs. Four studies, Fung et al. [44], Keeler et al. [31], Ng et al. [35], and Talboom et al. [33] reported no significant differences in blood transfusion needs between IV iron and standard of care. Laso-Morales et al. [34] also reported no significant differences in RBC transfusion needs in patients with CRC. However, it is important to note that this study compared two different IV iron products.

Six non-randomized studies reported changes in RBC transfusion needs. Of the studies that compared IV iron supplementation to oral iron supplementation, Calleja et al. [36] reported that patients in the IV iron group required less RBC transfusions when compared to patients in the oral iron group (9.9% vs. 38.7%; OR: 5.9. 95% CI: 2.9–11.1, *p*<0.001). In contrast, Laso-Morales et al. [48] reported higher transfusion needs in the IV iron group compared to the oral iron comparator group (15% vs. 4%, *p* = 0.040), however, the prevalence and severity of anemia (as indicated by Hb levels on day of surgery) were higher amongst those in the IV iron group. Furthermore, Quinn et al. [47] reported that prior to the introduction of the anemia management intervention, patients with anemia were 17 times more likely to require perioperative RBC transfusions. In addition, postoperative RBC transfusion rates were 38% in patients who received oral iron or no specific treatment, compared to 0% in patients whose anemia had been corrected by IV iron, and 3.5% in patients without anemia [47]. Kangaspunta et al. [43], Ploug et al. [46], Titos-Acros [40] reported no difference in RBC transfusion needs between the IV iron group and no IV iron group in their investigations.

### Patient health related outcomes

Four randomized studies collected data on patient health related outcomes. Laso-Morales et al. [34] reported no significant differences between LOS between the IV FCM group and the IV IS group, but did note that the infection rate was lower in the IV FCM group (9.8% vs. 37.2%). Ng et al. [35] reported a marked increase in QoL parameters such as physical and emotional well-being, as well as anemia-specific QoL, with total scores for these indices exceeding the minimum clinically important difference (defined as a difference of one standard deviation from baseline), while no improvement was reported in patients receiving standard of care. In addition, Keeler et al. [32] reported that 11 QoL components (e.g., physical and functional well-being, self-care, pain and disability, general health, etc.) increased by a clinically significant margin in the IV iron group, compared to only one component showing an increase in the oral iron group. Furthermore, patients in the IV iron group had higher median total scores (168, IQR:160–174 vs. 151, IQR:132–170) in the FACT–An than the oral iron group at the time of the outpatient appointment (2–3 months postoperatively) [.] Talboom et al. [33] reported no significant differences in postoperative complications and LOS between patients with CRC who received treatment with IV iron compared to patients receiving oral iron.

Eight non-randomized studies captured data on various health outcomes. Calleja et al. [36] reported that the IV iron group had a significantly shorter mean length of hospital stay compared to the no-IV iron group (8.4±6.8 days vs. 10.9±12.4 days; *p*<0.001). In addition, Calleja and colleagues [36] reported no adverse events (e.g., deaths, hypersensitivity, or other serious reactions) and there was no difference in post-surgical complications (e.g., suture dehiscence, paralytic ileus, hemoperitoneum, rectal bleeding, thromboembolism, etc.) at 30 days postoperatively [36]. Kangaspunta et al. [43] reported that patients with colon cancer treated up to 60 days preoperatively with 500–1000 mg of IV FCM had less post-operative complications (33.9% vs. 45.9%, *p* = 0.045), and no difference in LOS, 30- and 90-day mortality between the two groups. Laso-Morales et al. [48] reported no significant differences in postoperative infections, LOS, and complication rates between the two groups, but did report a significantly lower rate of postoperative infection in patients receiving IV iron compared to standard care patients (18% vs. 29%; *p* = 0.018). Ploug et al. 2022 [46] reported higher rates of surgical complications (25% vs. 8%; p = 0.01) in patients with CRC who received IV iron isomaltoside compared to patients who did not receive IV iron treatment. Ploug et al. 2022 [46] also reported no significant difference in LOS (p = 0.74) between the treatment and control group in their investigation. Quinn et al. [47] reported morbidity rates as similar across all groups, but did not provide the data. In addition, LOS was longer for patients with uncorrected anemia compared to patients with corrected anemia (13.2 days vs. 7.2 days; *p* = 0.019) (47). Wilson et al. [41] treated patients with CRC with 1000–2000 mg of IV FCM or iron (III) maltoside and found no significant difference in 1-, 3-, and 5-year OS between the IV iron and non-IV iron groups. Ploug et al. 2023 [45] reported that five-year recurrence of CRC was 13.5% in the IV iron group vs. 16.7% in the control group, and found that postoperative mortality rates (5 year follow up period) was 13.5% in the IV iron group compared to 2.8% in the control group, but, these claims were not tested for statistical significance.

## Discussion

Each included study was used to draw conclusions about the following key findings: Hb levels, RBC transfusion needs, iron parameters and patient QoL.

Sixteen studies included in this review evaluated Hb response among patient populations at varying timepoints. Five studies found no significant difference in Hb levels between the IV iron groups and the comparator groups whereas ten studies found a statistically significant increase in Hb levels among the groups receiving IV iron treatment. In contrast, one study reported lower mean Hb levels at discharge among patients with colon cancer receiving IV iron treatment when compared to the group receiving usual care [40]. Titos-Arcos *et al*., suggest that this finding is due to bone marrow in patients physiologically requiring more time to increase Hb levels postoperatively [40].

Eleven studies assessed the need for RBC transfusion in patients receiving IV iron compared to those receiving standard care. Two studies found a significant decrease in blood transfusion rates in groups receiving IV iron. In contrast, one study expected no significant differences in RBC transfusion rates between the study groups due to the use of two forms of active iron treatment, which would influence the need for blood transfusions [34]. The other eight studies found no significant difference in transfusion rates between study groups, despite increases in Hb levels in IV iron treatment groups. Keeler *et al*. explain this outcome as an insufficient duration of preoperative IV iron therapy which could resultantly influence transfusion rates [31]. Moreover, Ploug *et al*. found that 75% of RBC transfusions given to patients during the duration of the study were delivered in patients with Hb levels above the amount outlined in the transfusion guidelines, suggesting the need for more restrictive transfusion practices to avoid unnecessary RBCTs [46]. Other studies found no significant difference in RBC transfusion rates due to discrepancies at recruitment in which the need for transfusion was already low at recruitment among the groups or comparator arms had higher preoperative Hb levels, reducing the need for transfusions. Further research should address these limitations and have transfusion rates as a primary endpoint to produce more definitive findings on RBC transfusions.

A total of twelve studies analyzed a variety of patient health outcomes. Across these studies, findings generally revealed no significant difference in LOS, post-operative complications, and OS between IV iron groups and control groups. Two RCT studies measured an increase in QoL in the groups receiving IV iron treatment and two non-randomized studies revealed a decrease in post-operative infections in IV iron groups. Ploug *et al*., found higher rates of surgical complications in the group receiving IV iron treatment however, the two groups had similar OS and LOS rates.

A systematic review by Jones *et al*. reveals an improvement in Hb levels when patients with anemia who had surgery are treated with IV FCM [49]. All 10 RCT studies analyzed in the review found an improvement in Hb concentration from baseline to the end of the study in both the preoperative FCM (Hb concentration increase from 1.3 g/dL to 4.7 g/dL) and postoperative FCM setting (Hb concentration increase from 1.7 g/dL to 3.2 g/dL) (49). Moreover, a retrospective study by Cancado *et al*., evaluated the effects of administering IV iron sucrose (IS) infusions in an IDA (as defined by WHO guidelines) patient population by providing patients with a weekly dose of 200 mg IS until patients received a total iron dose (calculated by weight and Hb levels of the patient) or when they had a Hb concentration of greater than 14.0 g/dL [50]. Hb concentration in patients increased significantly between the baseline and end of study (Mean change: 3.29 g/dL (women) and 4.58 g/dL (men)) [50]. These results coincide with the findings of this review as most of the studies show a significant increase in Hb levels in patients receiving IV iron treatment opposed to other iron supplementation methods. These findings reveal the strong efficacy of IV iron in effectively increasing Hb levels in patients with IDA.

Hallet *et al*. systematically reviewed four studies to assess the effects of perioperative iron supplementation on RBC transfusion needs in patients undergoing elective GI surgeries [51]. The study found that although fewer patients required transfusions when given iron supplementation, the observations were statistically insignificant [51]. However, the findings of their systematic review may be underpowered, as the results were based on four studies with small sample sizes which may not provide an accurate effect estimate of iron supplementation [51]. Based on our systematic review findings, most studies that evaluated RBC transfusion rates as an outcome of interest found no significant difference between groups. However, a multicenter cohort study by Calleja *et al*. found that patients in the group receiving IV FCM (n = 111) needed less RBC transfusions than patients receiving oral iron supplementation (n = 155) (OR: 5.9, 95% CI: 2.9–11.1, p<0.001) [36]. Therefore, additional studies need to be conducted to assess the impact of IV iron and comparator iron types on blood transfusion rates to validate and strengthen the current evidence available. Furthermore, valid clinical endpoints, such as Hb rise, effective iron repletion, less RBC transfusion, morbidity, QoL and hospital length of stay for inpatient assessments, should be assessed in these studies.

Iron parameters were another key factor explored in studies evaluating the efficacy of IV iron treatment in IDA correction. Jones *et al*. found that in the preoperative setting, IV iron intervention revealed a 15–35% increase in TSAT levels from baseline and an increase in serum ferritin levels from 19 μg/L at baseline to 229–558 μg/L [49]. Additionally, they found that in the postoperative setting, there was a 7.2–20% increase in TSAT and a serum ferritin increase from 19 μg/L to 114–571 μg/L [49]. In our review, seven studies assessed iron parameters (serum ferritin and TSAT) to evaluate the difference in these biomarkers after treating patients with IV iron. All studies found a statistically significant increase in ferritin and TSAT levels of patients receiving IV iron compared to comparator groups. However, a limitation to these study findings is the use of serum ferritin as an indicator of IDA improvement in cancer patients. Ferritin levels are found to be elevated in the cancer patient population due to the cancer’s inflammatory nature [52]. The elevated levels of serum ferritin in cancer patients could be due to the abnormal production and release of ferritin from tumour cells [53]. Thus, the sensitivity of ferritin as a prognostic value for iron deficiency is low and studies should refrain from using this parameter as an indicator of iron levels in cancer patients.

QoL is an important factor that is often overlooked when treating IDA and assessing patient performance in an oncology population [36]. A review written by Strauss and Auerbach evaluated the importance of using validated patient reported outcome tools (FACT measurement system) to assess QoL [34]. They found IV iron (IV ferric gluconate) to be the most effective treatment for IDA in cancer patients which resulted in an improvement in patient FACT scores [34]. One RCT assessed in their review by Henry *et al*. looked at the impact of IV iron on FACT-Fatigue scores in patients with anemia receiving chemotherapy [36]. All patients in the trial received epoetin alfa once a week for four weeks, then an adjusted dose based on their protocol [36]. These patients were randomized into three groups: no iron (ESA alone), oral iron sulfate with ESA, or IV ferric gluconate with ESA [36]. Researchers found that patients receiving IV ferric gluconate reported a significant improvement in the FACT-Fatigue scale (MID = 3) compared to patients receiving oral iron or no iron [36]. In our systematic review, we found that the information on QoL specifically in patients with GI cancer was limited. Only two studies evaluated QoL as an outcome of interest. Keeler *et al*., found that the QoL Fact-An scores were higher in patients receiving IV iron compared to oral iron (FACT-An total score (oral iron 151 (132–170 [69–183]); IV iron 168 (160–174 [125–186]); p = 0.005))) [32]. However, these conclusions may not be meaningful due to the small sample size (n = 116) of the study [32]. More QoL research should be conducted in the GI cancer population to gain a stronger understanding of QoL improvement when given iron supplementation.

The strengths of this systematic review include the comprehensive and inclusive search strategy that was approved by an oncologist specializing in GI cancers, as well as a hematologist. This review assessed a total of 2,363 studies across five databases with no restrictions to language or publication types. Two authors (SN and NS) independently screened the eligibility of each of the studies to minimize selection bias. Moreover, the two authors (SN and NS) conducted separate data extractions for studies that passed the initial screening phase to ensure all pertinent information was collected and all studies followed the eligibility criteria of the review. Furthermore, the quality of both RCTs and non-RCTs were assessed using Cochrane’s RoB 2 and ROBINS-I tools. The risk assessment revealed the limitations of the studies included in this systematic review and helped curate a thorough understanding of the missing findings in current publications.

This systematic review presents some limitations. Due to the varying interventions, comparators, populations, clinical endpoints, iron formulation, dosing schemes and settings evaluated in each RCT, the results of the review produced heterogeneous findings and thus, made it difficult to conduct a meta-analysis. Additionally, the studies assessed in this review used different time points of data collection, included patients undergoing different surgical approaches, and different cancer types, making it difficult to make valid comparisons between findings. Some studies also assessed outcomes that did not provide meaningful conclusions, such as the use of serum ferritin as a prognostic parameter in this patient population. Moreover, a majority of studies in this review were potentially underpowered due to their small sample sizes. This review was not registered through PROSPERO or any other systematic review database, which is an additional limitation.

Overall, to strengthen the findings in this field, research should be conducted using larger sample sizes to validate findings prior to making conclusive statements on the efficacy of the intervention being used. Additionally, research evaluating the efficacy of IV iron should use iron parameters that are solely influenced by iron intake in patients with IDA (eg. Hb levels, RBC transfusion rates, serum iron, and TSAT) to ensure findings are resultant of the type of iron patients receive opposed to co-factors such as chemotherapy. More studies should also follow patients for a longer duration of time to assess the long-term impacts of IV iron on QoL and other important outcomes such as survival in this patient population. Another important consideration future researchers should incorporate in their research is the drug availability of IV iron compared to oral iron and how physicians can implement an IV iron infusion center in a feasible manner, especially as there are newer IV iron formulations (e.g., ferric derisomaltose) requiring shorter infusion times.

The findings of this systematic review reveal the importance of addressing the prevalence of IDA in a GI oncology patient population. Patients should receive timely diagnosis and management of ID prior to undergoing cancer treatments to avoid postoperative complications related to anemia, including myelosuppressive chemotherapy dose reductions, and to improve overall QoL. More research in this field will help create an international set of guidelines to ensure best clinical practices to improve IDA in patients with GI malignancies.

## Supporting information

S1 Appendix (DOCX)
