# Supplementary material for: Intravenous iron and iron deficiency anemia in patients with gastrointestinal cancer: A systematic review
Source: PLoS One. 2024 May 22;19(5):e0302964. doi: 10.1371/journal.pone.0302964 (PMC11111077; doi:10.1371/journal.pone.0302964)
Supplement: S1 Appendix — (DOCX) [file pone.0302964.s001.docx]

**Appendix**

*Full Search Strategy (no restrictions)*

(functional iron deficiency OR functional iron deficient anemia OR absolute iron deficiency OR absolute iron deficient anemia OR cancer related anemia OR chemotherapy-induced anemia OR chemotherapy induced anemia OR radiotherapy induced anemia OR radiotherapy-induced anemia OR anemia OR anaemia OR iron deficien* anemia OR cancer-related anemia OR microcytic anemia) AND (treatment OR therapy OR chemotherapy) AND (gastric cancer OR gastroesophageal cancer OR gastrointestinal cancer OR adenocarcinoma OR gastr* neoplasm OR gastr* carcinoma OR colo* cancer OR colo* neoplasm OR colo* carcinoma OR stomach cancer OR stomach neoplasm OR stomach carcinoma) AND (ESA OR ESA therap* OR erythropoiet* stimulating agents OR intravenous iron OR IV iron OR iron replacement OR iron supplement* OR parenteral iron OR iron sequestration OR myelosuppression OR iron studies OR serum iron OR total iron binding capacity OR transferrin saturation)
